# Effects of dexmedetomidine on smooth emergence from anaesthesia in elderly patients undergoing orthopaedic surgery

**DOI:** 10.1186/s12871-015-0127-4

**Published:** 2015-10-07

**Authors:** Dong Jun Kim, Sang Hun Kim, Keum Young So, Ki Tae Jung

**Affiliations:** 1 Department of Anesthesiology and Pain Medicine, Chosun University Hospital, 365 Pilmun-daero, Dong-gu, Gwangju, 501-717 South Korea; 2 Department of Anesthesiology and Pain Medicine, School of Medicine, Chosun University, 365 Pilmun-daero, Dong-gu, Gwangju, 501-717 South Korea

**Keywords:** Dexmedetomidine, Emergence agitation, Ricker’s agitation-sedation scale, Sevoflurane, Total intravenous anaesthesia

## Abstract

**Background:**

Intraoperative dexmedetomidine may decrease postoperative emergence agitation in elderly patients due to its sedative effect. In this study, we evaluated the effect of adjuvant dexmedetomidine on smooth emergence from anaesthesia after orthopaedic surgery in elderly patients.

**Methods:**

A total 115 patients (ASA I–II, aged over 65 years) were randomly allocated into four groups. Anaesthesia was maintained with either sevoflurane or total intravenous anaesthesia (TIVA) comprising propofol and remifentanil. Patients were also administered either dexmedetomidine (0.4 μg kg^−1^ hr^−1^; SD and TD) intraoperatively or normal saline (SN or TN) as a control. The bispectral index (BIS) score was maintained from 40–60 intraoperatively. All anaesthetics and dexmedetomidine were discontinued at surgical conclusion, and the recovery times (durations to a BIS = 60, 70, and 80; eye opening; and extubation) were measured. The mean arterial pressure, heart rate, Ricker’s agitation-sedation scale (RSAS), visual analogue scale (VAS) for pain, and incidences of emergence agitation and postoperative nausea and vomiting (PONV) were measured in the recovery room.

**Results:**

Dexmedetomidine significantly decreased the RSAS score in the SD and TD groups, and a calm state postoperatively occurred more frequently in these groups than in the control groups. The heart rate and incidence of emergence agitation were lower in the dexmedetomidine groups. Recovery time was higher in the SD group than in the SN group, and no significant differences occurred between the TN and TD groups. The VAS score was lower in the SD group than in the SN group, and the PONV did not differ regardless of the use of dexmedetomidine.

**Conclusions:**

Dexmedetomidine may be an effective intraoperative adjuvant for a reducing emergence agitation and smooth emergence from anaesthesia after orthopaedic surgery in elderly patients.

**Trial registration:**

Current Controlled Trials NCT01851005.

## Background

As the elderly population continues to increase, surgery of elderly patients is also on the rise. The risk of postoperative complications during recovery such as emergence agitation or delirium in the elderly is also increasing [1]. Emergence agitation/delirium, which have been used interchangeably in most of the literature [2], defined as a state of mild restlessness and mental distress and a restless or confused status after emergence from anaesthesia [2, 3]. This can be dangerous due to harmful behaviours by the patients such as injury, haemorrhage, and self-extubation [3]. The incidence of delirium in the elderly is highest after major orthopaedic surgery [4]. A recent study showed that dexmedetomidine reduced the incidence of emergence agitation in children after sevoflurane anaesthesia [5], and another study found that intraoperative adjuvant dexmedetomidine can improve the stability of recovery from anaesthesia [6]. Thus, in the present study, we investigated the effect of adjuvant dexmedetomidine on emergence from anaesthesia undergoing elective orthopaedic surgery recovery in elderly patients targeting smooth emergence.

## Methods

This study was approved by the Internal Review Board (IRB) of Chosun University Hospital and the Korean Food & Drug Administration (KFDA, 20130039565), and was registered at http://clinicaltrials.gov (registration number NCT01851005).

A total 115 patients at ASA class I-II, aged over 65 years, and scheduled for elective orthopaedic surgery were enrolled. Patients with the following conditions were excluded: severe heart disease with a New York Heart Association class > III, severe arrhythmia, uncontrolled hypertension or hypotension, hemodynamic instability, drug hypersensitivity, any cognitive deficiency, hepatic or renal compromise, infectious disease, and surgery lasting more than 3 h. Written informed consent was obtained from all patients after full explanation of the study.

Patients were randomly allocated into two groups according to anaesthetic method (sevoflurane or total intravenous anaesthesia, TIVA) using computer-generated random numbers. They were allocated again into four groups according to the use of dexmedetomidine. In the SN group (*n* = 28) and SD group (*n* = 27), anaesthesia was maintained with sevoflurane and a 50 % O_2_-air mixture. In the TN (*n* = 30) and TD (*n* = 30) groups, anaesthesia was maintained TIVA comprising propofol and remifentanil. In the SD and TD groups which were the experimental group, dexmedetomidine (Precedex® 100 mg/ml, Hospira, Inc., Rocky Mount, IL, USA) was administered at 0.4 μg kg^−1^ hr^−1^ after aesthetic induction until surgical conclusion [6]. Normal saline was administered at the same rate (0.1 ml kg^−1^ hr^−1^) as a control in the SN and TN groups which were the control group. Adjuvant drug was prepared by an independent nurse who was not involved in the management of the patients. Dexmedetomidine was diluted to a 50-ml volume, normal saline was prepared in a 50-ml syringe, and each drug was labelled as coded syringes. Thus, the anaesthesiologist and nurses who participated in anaesthesia and recovery were blinded to the adjuvant drugs.

All patients were administered midazolam (0.05 mg kg^−1^, intramuscularly) as a premedication 30 min before anaesthesia. Upon arrival to the surgical suite, the electrocardiogram (ECG), pulse oximetry for oxygen saturation (SpO2), non-invasive arterial pressure (NIBP), and bispectral index (BIS) (BIS monitor A-2000; Aspect Medical Systems, Norwood, MA, USA) were monitored. Anaesthesia was induced according to the following protocols. In the SN and SD groups, anaesthesia was induced with propofol at 1.5–2.0 mg kg^−1^ and manual mask ventilation was done with 3–4 vol% sevoflurane and 50 % O_2_-air mixture until adequate neuromuscular blocking was achieved. In the TN and TD groups, anaesthesia was induced with remifentanil and propofol based on a Minto and Marsh pharmacokinetic model using a TCI device (Orchestra® Base Primea, Fresenius-Vial, France). The targeted effect-site concentrations (Ce) of propofol and remifentanil for induction were 3 μg/ml and 2.5 ng/ml, respectively. Once the patient lost consciousness, rocuronium was administered at 1.0 mg kg^−1^ and endotracheal intubation was performed. During mechanical ventilation, the initial tidal volume was set at 8 ml/kg, and the tidal volume and frequency were adjusted to maintain the end-tidal CO_2_ between 4.6 and 5.3 kPa. Anaesthesia was maintained with sevoflurane or propofol-remifentanil and a 50 % O_2_-air mixture. During the maintenance of anaesthesia, the end-tidal concentration of sevoflurane and the Ce of propofol and remifentanil were adjusted to maintain the BIS score near 50 (range 40–60) and maintain the vital signs within 20 % of their baseline values. Those values were recorded before administrating the adjuvant drugs and at 5 min, 10 min, 15 min, 30 min, 60 min, and 120 min after infusion.

After anaesthetic induction, the adjuvant drugs (dexmedetomidine or normal saline) were infused. The mean arterial pressure (MAP) and heart rate (HR) were measured before administering the adjuvant drugs, 10 min and 30 min after infusion of adjuvant drugs, upon arrival in the recovery room, 15 min after arrival in the recovery room, and 30 min after arrival in the recovery room. When bradycardia (HR < 40 bpm), tachycardia (HR > 110 bpm), hypertension (MAP > 110 mmHg), or hypotension (MAP < 60 mmHg) were observed, atropine 0.5 mg, esmolol 10 mg, nicardipine 0.5 mg, ephedrine 5 mg, or phenylephrine 100 μg was administered as treatment, respectively.

At the end of surgery, fentanyl administration was started using patient controlled anaelgesia instrument (initial loading 0.9 μg kg^−1^; basal infusion, 0.625 μg kg^−1^ hr^−1^; intermittent bolus, 0.9 μg kg^−1^ hr^−1^; lockout time, 30 min) according to the hospital protocol before administration of reversal agents. Reversal agents (glycopyrrolate 0.004 mg kg^−1^ and neostigmine 0.02 mg kg^−1^) were administered, and the recovery from the neuromuscular block was confirmed using a train-of-four ratio. Administration of all anaesthetics and adjuvant drugs was discontinued, and disturbance or stimulation of the patients was avoided, except a verbal request to open their eyes. When the patients could breathe spontaneously, follow the command to open their eyes, and had a BIS score greater than 80, they were extubated and transferred to the recovery room. The recovery times were measured as follows: the duration from discontinuing all drugs to achieving a BIS score of 60, 70, and 80; the duration in minutes until the patients responded to the command of eye opening; and the duration to extubation [7, 8].

The patients were transferred to the recovery room, and the BP, HR, 11-point visual analogue scale for pain (0 = no pain and 10 = worst pain imaginable), and the Riker’s sedation-agitated scale (RSAS) were measured. The RSAS was measured three times at 15-min intervals according to the following scale: 1 = minimal or no response to noxious stimuli; 2 = arousal to physical stimuli but noncommunicative; 3 = difficult to arouse but awakens to verbal stimuli or gentle shaking; 4 = calm and follows commands; 5 = anxious or physically agitated but calms to verbal instructions; 6 = requires restraint and frequent verbal reminding of limits; and 7 = attempting to remove tracheal tube or catheters, or striking at staff [9]. Patients were classified according to the RSAS score (anxious or agitated, RSAS 5 to 7; calm, RSAS 4; and sedated, RSAS 1 to 3) [9]. Each patient’s maximum RSAS score, and the incidences of emergence agitation (scale ≥5) and dangerous EA (score = 7) were recorded [3, 6, 9]. The pain score was measured using the visual analogue scale (VAS) in the recovery room. The incidence of postoperative nausea and vomiting (PONV) was also measured by asking the presence of PONV to the patients. The duration of hospitalization in the recovery room was also measured.

### Statistical analysis

Sample size was calculated using “G*Power3” free software (available at: http://www.psycho.uni-duesseldorf.de/abteilungen/aap/gpower3). The effect size was 0.329, which was calculated based on a previous study in which the incidence of emergence agitation decreased from 52 to 28 % after dexmedetomidine infusion [6]. Using α = 0.05 with a power of 80 %, the total sample size was calculated at 108. After assuming a 10 % drop out rate, 30 patients were allocated to each group.

Statistical analyses were performed using SPSS 12.0 (SPSS, Inc., Chicago, IL, USA). The normality of distribution was assessed with the Kolmogorov-Smirnov test. Values are expressed as the mean (SD) or the number of patients (%). Parametric data (weight, recovery time, maximum RSAS score, and VAS) were analysed with the one-way ANOVA test; the Mann–Whitney U-test was used for post-hoc analysis. Non-parametric data (age, height, duration of surgery, duration of recovery room stay) were analysed using the Kruskal Wallis test. Categorical variables (gender, ASA class, type of surgery, and incidences of EA and PONV) were analysed by the Chi-square or Fisher’s exact test. A *P* < 0.0125 was considered statistically significant, accounting for a Bonferroni correction in comparison of four groups and a *P* < 0.05 was considered statistically significant in comparison of experimental groups and control groups. The change in the end-tidal concentration of sevoflurane and the Ce of propofol and remifentanil were analysed by repeated measures ANOVA, and post-hoc testing was performed using the turkey and t-test. A *P* < 0.05 was considered statistically significant between two groups.

## Results

**Fig. 1:**
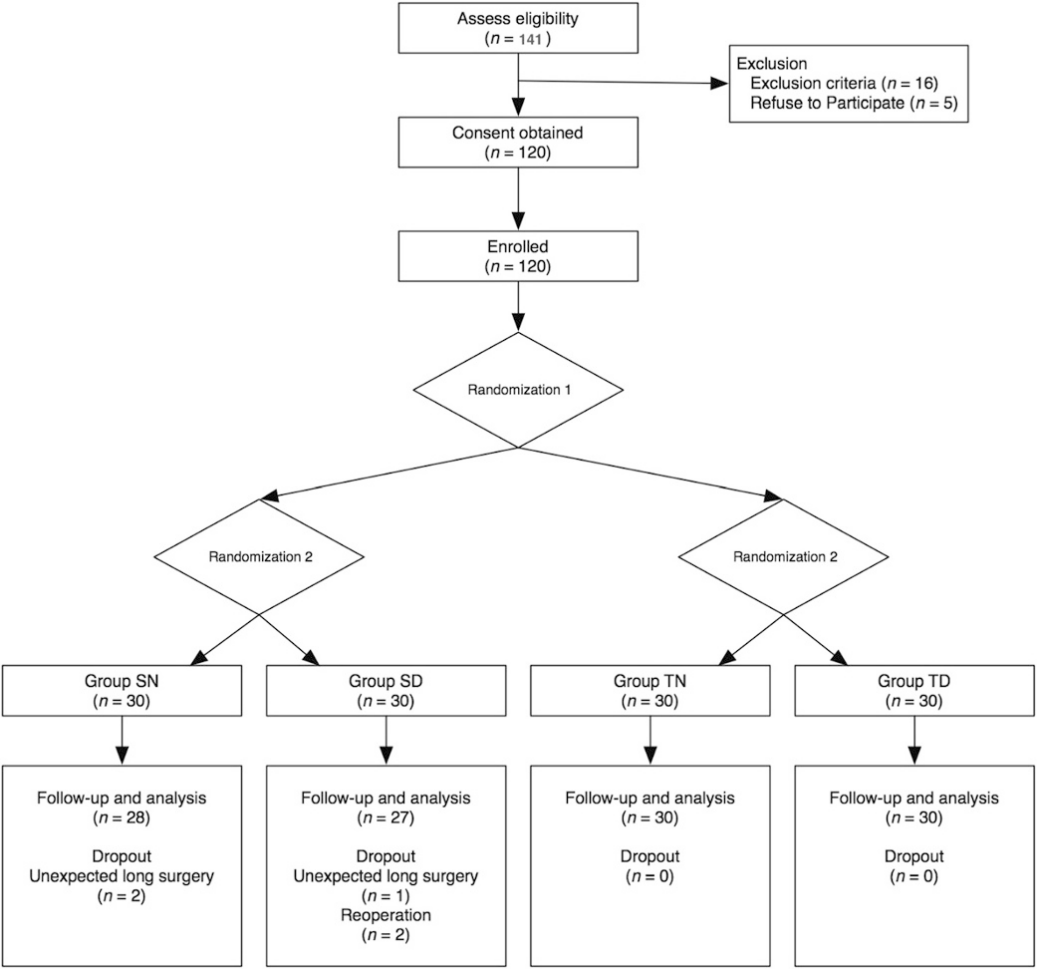
Consort flow diagram for the study. Group SN, inhalation anaesthesia using sevoflurane and normal saline administered as a control; Group SD, inhalation anaesthesia using sevoflurane and adjuvant dexmedetomidine; Group TN, TIVA using propofol and remifentanil and normal saline administered as a control; Group TD, TIVA using propofol and remifentanil, and adjuvant dexmedetomidine

A total 141 patients were assessed for eligibility, and 120 patients were enrolled. Two patients in the SN group and one patient in the SD group were excluded because the surgery lasted more than 180 min. One patient in the SD group was excluded because of reoperation due to postoperative haemorrhage. Finally, 115 patients were analysed (Fig. 1).

**Fig. 2:**
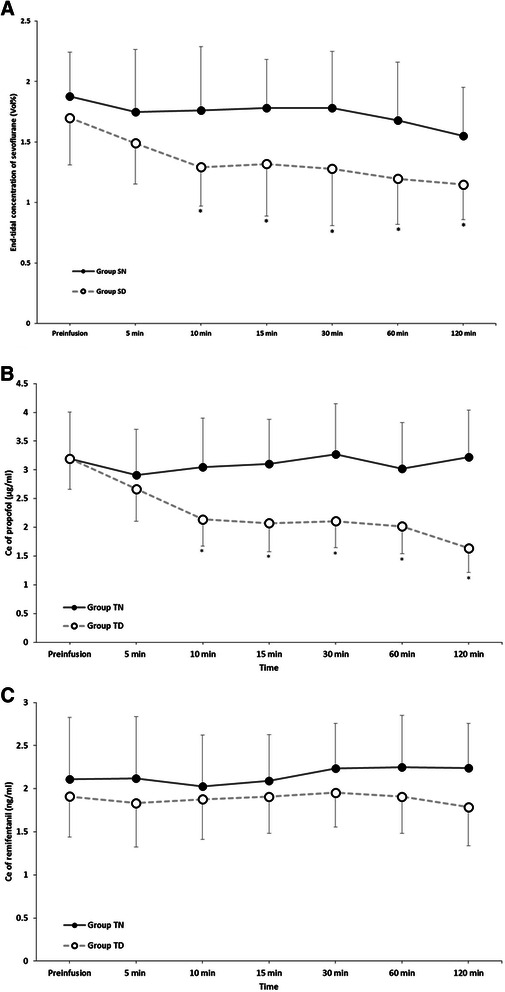
End-tidal concentration of sevoflurane (**a**), the effect site concentration of propofol (**b**), and the effect site concentration of remifentanil (**c**) during anaesthesia. Group SN, inhalation anaesthesia using sevoflurane and normal saline administered as a control; Group SD, inhalation anaesthesia using sevoflurane and adjuvant dexmedetomidine; Group TN, TIVA using propofol and remifentanil and normal saline administered as a control; Group TD, TIVA using propofol and remifentanil, and adjuvant dexmedetomidine; TIVA, total intravenous anaesthesia; Ce, effect site concentration. *P < 0.05 compared with the control group

There were no significant differences in the characteristic data, type of surgery, and duration of surgery between the groups (Table 1). During anaesthetic maintenance, the end-tidal concentration of sevoflurane was lower in the SD group than in the SN group at 10 min after administering dexmedetomidine (*P* < 0.05, Fig. 2a). The Ce of propofol in the TD group was less than that in the TN group at 10 min after administering dexmedetomidine (*P* < 0.05, Fig. 2b). However, the Ce of remifentanil did not show any significant differences between the TS and TD groups (*P* > 0.05, Fig. 2c).

**Table 1:** Patient characteristics, surgery type, and surgical duration

	*P* value (all groups)	Group SN (*n* = 28)	Group SD (*n* = 27)	*P* value (group SN vs. SD)	Group TN (*n* = 30)	Group TD (*n* = 30)	*P* value (group TN vs. TD)
Age (yr)	0.587	72.3 (6.2)	72.6 (4.3)	0.066	73.5 (7.2)	74.5 (6.5)	0.884
Gender (M/F)	0.977	8/20	9/19	0.873	8/22	10/20	0.791
Height (cm)	0.547	156.3 (8.4)	158.8 (7.5)	0.888	156.9 (7.8)	158.5 (9.0)	0.301
Weight (kg)	0.895	58.3 (8.2)	58.0 (9.7)	0.368	56.7 (10.4)	58.4 (11.2)	0.844
ASA class (I/II)	0.115	8/20	4/23	0.217	11/19	13/17	0.598
Type of surgery	0.656			0.747			0.980
Total hip replacement		4 (14.3)	2 (7.4)		4 (13.3)	4 (13.3)	
Total knee replacement		7 (25)	5 (18.5)		3 (10)	4 (13.3)	
Long bone fracture fixation		6 (21.4)	7 (25.9)		13 (43.3)	12 (40)	
Spinal surgery		11 (39.3)	14 (48.1)		10 (33.3)	10 (33.3)	
Duration of surgery (min)	0.717	116.7 (29.7)	115.7 (29.6)	0.684	115.3 (39.1)	107.0 (34.6)	0.386

Values are the mean (SD) or number (%). Group SN, inhalation anaesthesia using sevoflurane and normal saline administered as a control; Group SD, inhalation anaesthesia using sevoflurane and adjuvant dexmedetomidine; Group TN, TIVA using propofol and remifentanil and normal saline administered as a control; Group TD, TIVA using propofol and remifentanil, and adjuvant dexmedetomidine; *TIVA* total intravenous anaesthesia, *SD* standard deviation

**Fig. 3:**
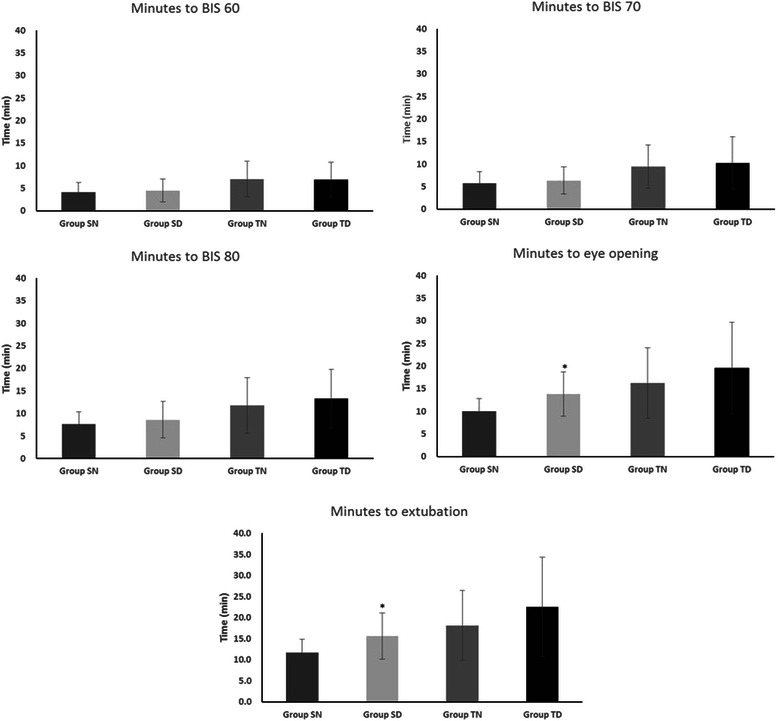
Recovery times following orthopaedic surgery. Group SN, inhalation anaesthesia using sevoflurane and normal saline administered as a control; Group SD, inhalation anaesthesia using sevoflurane and adjuvant dexmedetomidine; Group TN, TIVA using propofol and remifentanil and normal saline administered as a control; Group TD, TIVA using propofol and remifentanil, and adjuvant dexmedetomidine; TIVA, total intravenous anaesthesia; BIS, bispectral index. *P < 0.05 compared with SN

Recovery times are presented in Fig. 3. The duration to eye opening and extubation were longer in the SD group compared to those in the SN group (10.0 min vs. 13.8 min, and 11.7 min vs. 15.7 min, *P* = 0.001 and 0.004, respectively). However, there were no significant differences in the recovery times between the TIVA groups regardless of whether dexmedetomidine was used.

**Fig. 4:**
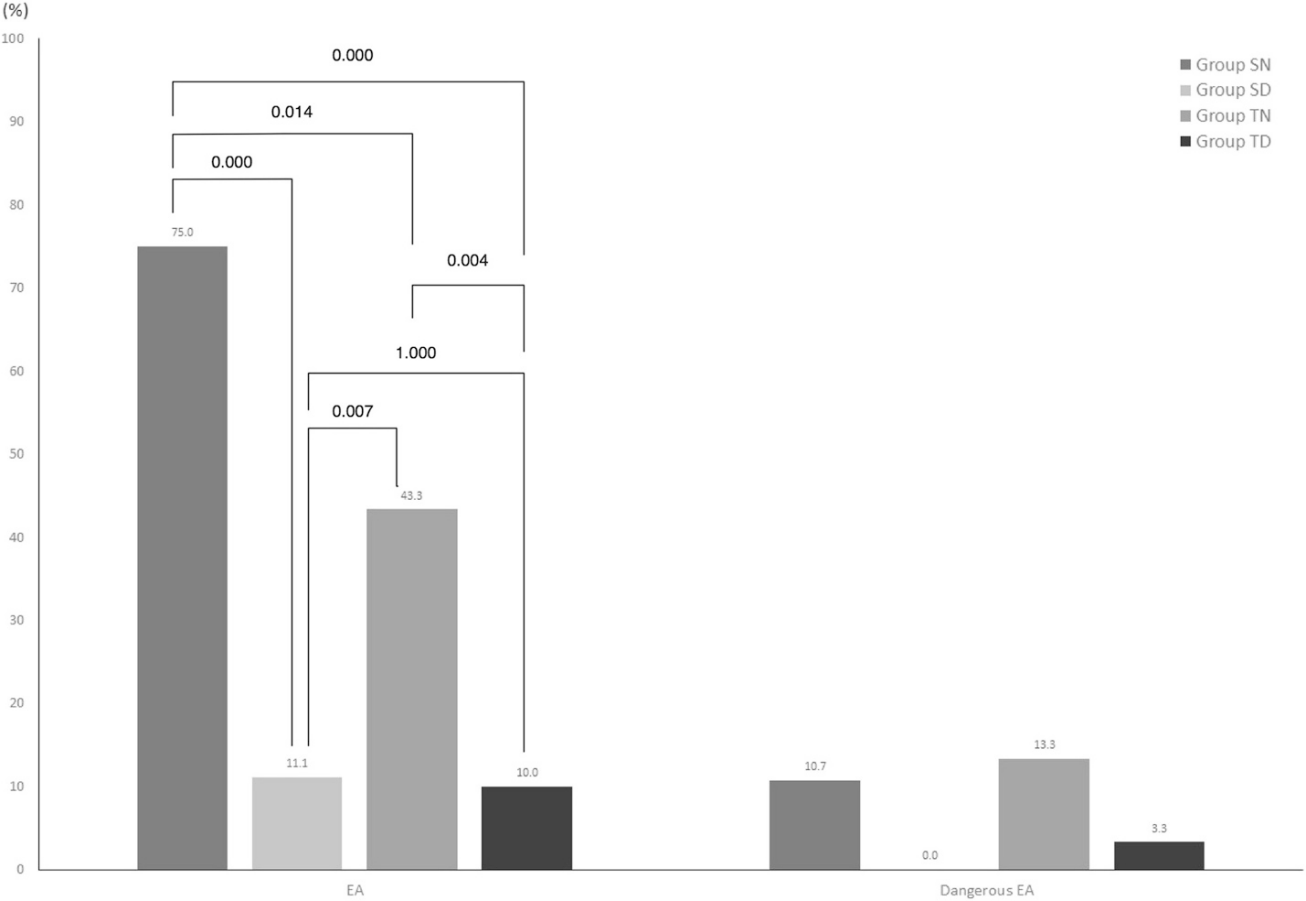
Incidence of emergence agitation and dangerous emergence agitation in the recovery room 30 min after arrival. Group SN, inhalation anaesthesia using sevoflurane and normal saline administered as a control; Group SD, inhalation anaesthesia using sevoflurane and adjuvant dexmedetomidine; Group TN, TIVA using propofol and remifentanil and normal saline administered as a control; Group TD, TIVA using propofol and remifentanil, and adjuvant dexmedetomidine; TIVA, total intravenous administration; EA, emergence agitation. Emergence agitation was defined as a Riker’s sedation-agitation scale greater than 5, and dangerous EA was defined as a score greater than 7

The maximum RSAS score differed significantly between the groups (*P* < 0.001, Table 3). The maximum scores were lower in the experimental groups compared to the control groups (5.4 ± 1.0 in group SN vs. 4.1 ± 0.3 in group SD, *P* < 0.001; 4.9 ± 1.2 in group TN vs. 4.2 ± 0.7 in group TD, *P* = 0.021). Patients were also categorized after arrival to the recovery room according to the RSAS (Table 2). Patients who were administered dexmedetomidine maintained RSAS score less than 5 (40.7 % in group SN vs. 25 % in group SD, P < 0.001; 36.7 % in group TN vs. 53.3 % in group TD, *P* = 0.013) lasting until 15 min into recovery (71.4 % in group SN vs. 88.9 % in group SD, *P* = 0.013; 66.7 % in group TN vs. 90 % in group TD, *P* = 0.021). Patients administered dexmedetomidine showed a lower incidence of emergence agitation compared to the control patients (*P* < 0.001). The incidence of emergence agitation was 11.1 % and 10.0 % in the SD and TD groups, and 75.0 and 43.3 % in the SN and TN groups, respectively. The incidences of dangerous emergence did not differ between the groups (Fig. 4).

**Table 2:** Patient categorization according to the Riker’s sedation-agitation scale in the recovery room

	Category	*P* value (all groups)	Group SN (*n* = 28)	Group SD (*n* = 27)	*P* value (group SN vs. SD)	Group TN (*n* = 30)	Group TD (*n* = 30)	*P* value (group TN vs. TD)
At arrival	Sedated	<0.001	1 (3.6)	13 (48.1)	<0.001	6 (20.0)	11 (36.7)	0.013
	Calm		7 (25.0)	11 (40.7)		11 (36.7)	13 (53.3)	
	Agitated		20 (71.4)	3 (11.1)		16 (43.3)	3 (10.0)	
After 15 min	Sedated	0.005	0 (0.0)	2 (7.4)	0.013	0 (0.0)	1 (3.3)	0.021
	Calm		20 (71.4)	24 (88.9)		20 (66.7)	27 (90.0)	
	Agitated		8 (28.6)	1 (3.7)		10 (33.3)	2 (6.7)	
After 30 min	Sedated	0.196	0 (0.0)	0 (0.0)	0.193	0 (0.0)	0 (0.0)	0.097
	Calm		24 (82.1)	26 (96.3)		26 (86.7)	29 (96.7)	
	Agitated		5 (17.9)	1 (3.7)		4 (13.3)	1 (3.3)	

Values are the number (%). Patients were categorized as agitated, RSAS 5 to 7; calm, RSAS 4; and sedated, RSAS 1 to 3 while in the recovery room postoperatively. Group SN, inhalation anaesthesia using sevoflurane and normal saline administered as a control; Group SD, inhalation anaesthesia using sevoflurane and adjuvant dexmedetomidine; Group TN, TIVA using propofol and remifentanil and normal saline administered as a control; Group TD, TIVA using propofol and remifentanil, and adjuvant dexmedetomidine; *TIVA* total intravenous anaesthesia, *RSAS* Riker’s sedation-agitation scale

**Fig. 5:**
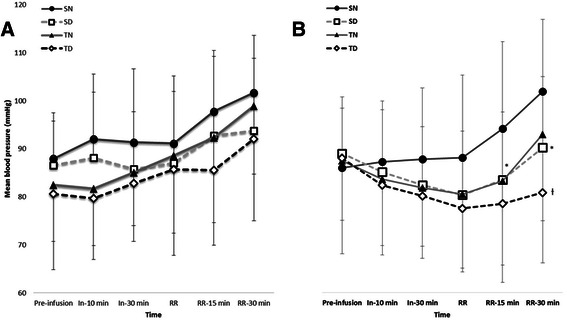
Hemodynamic changes during infusion of adjuvant drugs and during anaesthetic emergence. **a** Mean arterial pressure and (**b**) heart rate measured. Pre-infusion, before infusion of adjuvant drugs; In-10 min, 10 min after infusion of adjuvant drugs; In-30 min, 30 min after infusion of adjuvant drugs; RR, at arrival to recovery room;, RR-15 min, 15 min after arrival to recovery room; RR-30 min, 30 min after arrival to recovery room. Group SN, inhalation anaesthesia using sevoflurane and normal saline administered as a control; Group SD, inhalation anaesthesia using sevoflurane and adjuvant dexmedetomidine; Group TN, TIVA using propofol and remifentanil and normal saline administered as a control; Group TD, TIVA using propofol and remifentanil, and adjuvant dexmedetomidine. *P < 0.05 compared with the SN group. †P < 0.05 compared with the TN group

There were no significant differences in MAP during infusion of adjuvant drugs and between the dexmedetomidine and control groups (*P* > 0.05, Fig. 5). The HR was lower in the SD group compared to that in the SN group 15 min after the patients arrived in the recovery room. The HR was also lower in the TD group TD compared to that in the TN group 30 min after arrival to the recovery room.

The VAS score at the recovery room in the SD group was less than that in the SN group (*P* = 0.011), however, there was no significant differences between group TN and TD. The incidence of PONV differed only according to the anaesthetic method (Table 3). There were no significant differences between the SN and SD groups (*P* = 0.646), and between the TN and TD groups (*P* = 0.640). There were no differences in the duration of recovery room stay between the groups (Table 3).

**Table 3:** Maximum scores for the Riker’s sedation-agitation scale, visual analogue score, and incidence of postoperative nausea and vomiting

	*P*-value (all groups)	Group SN (*n* = 28)	Group SD (*n* = 27)	*P* value (group SN vs. SD)	Group TN (*n* = 30)	Group TD (*n* = 30)	*P* value (group TN vs. TD)
Maximum RSAS	<0.001	5.4 (1.0)	4.1 (0.3)	<0.001	4.9 (1.2)	4.2 (0.7)	0.041
VAS	0.020	6.6 (1.9)	5.3 (1.9)	0.011	5.7 (2.1)	5.1 (1.6)	0.246
PONV	0.003	11 (39.3)	9 (34.6)	0.654	3 (10.0)	2 (6.7)	0.732
Duration of recovery room stay (min)	0.567	42.1 (6.7)	41.5 (6.1)	0.534	42.0 (7.3)	43.2 (7.3)	0.276

Values are the mean (SD) or number (%). Group SN, inhalation anaesthesia using sevoflurane and normal saline administered as a control; Group SD, inhalation anaesthesia using sevoflurane and adjuvant dexmedetomidine; Group TN, TIVA using propofol and remifentanil and normal saline administered as a control; Group TD, TIVA using propofol and remifentanil, and adjuvant dexmedetomidine; *RSAS* Riker’s sedation-agitation scale, *VAS* visual analogue score, *PONV* postoperative nausea and vomiting

## Discussion

In this study, dexmedetomidine significantly decreased the RSAS score, the incidence of emergence agitation, and the patients in the dexmedetomidine groups remained calm state after surgery more frequently than did the patients in the control groups. These results suggest that dexmedetomidine (0.4 μg kg^−1^ hr^−1^) can be used as an effective adjuvant drug intraoperatively to ensure a smooth recovery after orthopaedic surgery in elderly patients.

During emergence from anaesthesia, elderly patients may experience psychological dysfunction such as delirium, agitation, confusion, or cognitive dysfunction, which is associated with postoperative morbidity [3, 10, 11]. Although the mechanism of postoperative psychological dysfunction is unclear and multifactorial, postoperative pain [12], hypoxia [13], opioid use [14], and anaesthetic technique [15] may encourage the development of postoperative delirium. Elderly patients who are undergoing orthopaedic surgery are at a particularly high risk [4].

Recent studies showed that dexmedetomidine decreased emergence agitation in children [5], and decreased postoperative delirium after cardiac surgery [16] and nasal surgery [6]. Dexmedetomidine is a highly selective α_2_-receptor agonist that has a sedative effect [17]. It may also affect the presynaptic noradrenergic transmission, which may decrease the potential causative factors of delirium [16, 18]. Dexmedetomidine also provides analgesia without causing significant respiratory depression [19]. It may help reduce opioid use while protecting against hypoxia due to hypoventilation in during anaesthetic recovery, and consequently may reduce the development of delirium. However, the sedative effects of dexmedetomidine may also influence recovery after anaesthesia when used as an adjuvant drug.

It has been shown that adjuvant dexmedetomidine during general anaesthesia decreases the sevoflurane or propofol required to maintain an adequate depth of anaesthesia [20–22]. Consistent with a study by Patel [20], in the present study, the end-tidal concentration of sevoflurane after 60 min was lower in the SD group (1.20 ± 0.38) compared to the SN group (1.68 ± 0.48, P < 0.001). According to the previous studies, dexmedetomdine decreased effective end-tidal concentration of isoflurane and effective plasma concentration of propofol for abolishing motor or verbal responses [23]. In case of motor response, end-tidal isoflurane concentration at which 50 % of subjects responded was decreased from 1.048 to 0.722 % when plasma concentration of dexmedetomidine was maintained as 0.3 ng/ml. According to the pharmacokinetic simulation using pharmacokinetic profiles of Korean subjects [24], plasma concentration of demedetomidine reached 0.1 ng/ml at about 10 min after continuous infusion (0.4 μg kg^−1^ hr^−1^). At this point, end-tidal concentration of sevoflurane started to became lower in the group SD than group SN (1.29 in group SD vs. 1.76 in group SN) in this study. Although calculated plasma concentration of demedetomidine is less than that of previous study, the subjects of this study were elderly, not healthy adult. Thus, we thought low dose infusion of dexmedetomidine (01 ng/ml of plasma concentration) could be effective to reduce the end-tidal concentration of sevoflurane during anaesthesia. According to a study conducted by Le Guen, when dexmedetomidine was administered, the propofol dosage decreased approximately 29 % during anaesthetic maintenance; however, there was no significant difference in the remifentanil dosage [22]. These results are consistent with our findings. The effect site concentration of propofol was also lower in the TD group compared to the TN group after 10 min (mean 3.11 vs. 2.26, respectively, *P* = 0.026), but the effect Ce of remifentanil was not significantly different (*P* = 0.974). Moreover, there were no significant differences in the postoperative VAS scores. This finding may be because dexmedetomidine produces analgesia through the α_2_-receptor, which is less effective than opioids receptors [25]. Dexmedetomidine acts primarily as a sedative rather than an analgesic [22].

The recovery time increased in groups using TIVA compared to those using sevoflurane. Propofol appears to have a prolonged effect in the elderly because of its lipid solubility and the proportion of fat in the total body weight in elderly patients [8]. A significant difference in the recovery time between the dexmedetomidine and control groups was only observed prior to eye opening and extubation between the SN and SD groups. However, there was a remarkable difference observed in a previous study conducted by Ohtani and others [7]. They reported that dexmedetomidine delayed the emergence from propofol anaesthesia and suspected that the pharmacodynamic interaction between dexmedetomidine and propofol may have delayed emergence from anaesthesia [7]. In this study, we adjusted the Ce of propofol according to the BIS score; thus, dexmedetomidine decreased the administered propofol. Consequently, there were no significant differences in the recovery time between the TN and TD groups.

Kim and others reported that the intraoperative infusion of dexmedetomidine provided smooth emergence and decreased agitation after nasal surgery [6], which agrees with our present results. In the current study, dexmedetomidine decreased emergence agitation (75 % in the SN group vs. 11.1 % in the SD group, *P* < 0.001; 43.3 % in the TN group vs. 10.1 % in the TD group, *P* = 0.004), but there were no significant difference in the incidence of dangerous emergence agitation. When patients arrived at the recovery room after surgery, more patients remained in a calm state when dexmedetomidine was administered (25.0 % in the SN group vs. 40.7 % in the SD group, *P* < 0.001; 36.7 % in the TN group vs. 53.3 % in the TD group, *P* = 0.013). However, none of the patients were sedated after 30 min. Though it remains uncertain, the sedative property of dexmedetomidine and the pharmacodynamic interaction between dexmedetomidine and anaesthetics may affect the sedative state after anaesthetic recovery [7, 26]. Further evaluation of the interaction between adjuvant dexmedetomidine and anaesthetics is needed.

Similarly, a study conducted by Kim and others reported that the postoperative heart rate decreased in patients administered dexmedetomidine [6]; however, there were no statistical differences in the MAP between the experimental and control groups. Potentially, the timing of measurement may have generated these different outcomes. Kim and others measured the MAP and HR when the surgery was complete and after extubation [6]. By contrast, we measured these parameters later when the patients arrived in the recovery room. Regardless, the ability of dexmedetomidine to control the increase in heart rate during recovery is attractive.

There are several limitations in this study. First, the gender distribution is uneven. Although gender has no known effect on the development of emergence delirium [4], it may potentially affect the recovery time and other characteristics. Second, we did not evaluate the long-term effect of dexmedetomidine on postoperative delirium. We focused solely on recovery characteristics and smooth emergence after anaesthesia with dexmedetomidine administration. However, postoperative delirium can develop up to 3 days postoperatively [4]; therefore, further evaluation employing long-term follow up is needed. Third, we did not administer a loading dose of dexmedetomidine. When using dexmedetomidine to induce sedation, an initial loading dose is required, but this protocol is associated with sudden hemodynamic changes [27]. When we contacted with IRB and KFDA for approval of the protocol of this study, the best concern was that the dexmedetomidine is not an anaesthetic drug and there is no guarantee of safety when administered as conventional method with other anaesthetic drugs. However, adjuvant dexmedetomidine administered intraoperatively without an initial loading effectively reduced emergence agitation according to a previous study [6]. In addition, the primary outcome was emergence agitation and smooth emergence from anaesthesia, not the hemodynamic response during anaesthesia. Thus, we used the protocol which does not used loading dose of dexmedetomidine and slow rate of infusion (0.4 μg kg^−1^ hr^−1^) as employed previously [6]. Fourth, we did not count the use of haemodynamic drugs, which was methodological error.

## Conclusions

The administration of dexmedetomidine (0.4 μg kg^−1^ hr^−1^) decreased the emergence agitation after orthopaedic surgery in elderly patients. These results may support the adjuvant use of dexmedetomidine for smooth emergence from anaesthesia and further evaluation of the potential of dexmedetomidine to reduce long-term cognitive dysfunction in elderly patients.

## Abbreviations

TIVA: Total intravenous anaesthesia
BIS: The bispectral index
MAP: mean arterial pressure
RSAS: Ricker’s agitation-sedation scale
VAS: Visual analogue scale
EA: Emergence agitation
PONV: Postoperative nausea and vomiting
ANOVA: Analysis of variance
